# Supplementary material for: Spatiotemporal chaotic unjamming and jamming in granular avalanches
Source: Sci Rep. 2015 Jan 30;5:8128. doi: 10.1038/srep08128 (PMC4311237; doi:10.1038/srep08128)
Supplement: Supplementary Information — Spatiotemporal Chaotic unjamming and jamming in granular avalanches [file srep08128-s1.pdf]

Supplementary Material for "Spatiotemporal Chaotic unjamming and jamming in granular avalanches"

Ziwei Wang[1] and Jie Zhang[2,3,*]
[1]Zhiyuan College & [2]Institute of Natural Sciences & [3]Department of Physics and Astronomy, Shanghai Jiao Tong University, Shanghai 200240, China

I. A COMMENT ON THE LYAPUNOV VECTOR IN PHASE SPACE

In the figure 1 of the main text, we have shown that there is a strong spatial correlation between δu and v at the unjamming transition, which suggests that a particle will gain more kinetic energy if the particle is more unstable. At the jamming transition, the correlation becomes weaker. In the above measurement of δu, the deviation between the actual trajectory and the ideal trajectory is computed in real space not in phase space, i.e. no contribution from the velocity part is considered. It is intuitive that considering the contribution of the velocity will only increase the spatial correlation between the two quantities as shown in the figure 1 of the main text. We also note that adding contributions from the velocity part will make no qualitative difference in the evolution of the global Lyapunov vector $\delta U(t)$ of the system as shown in Fig. 1.

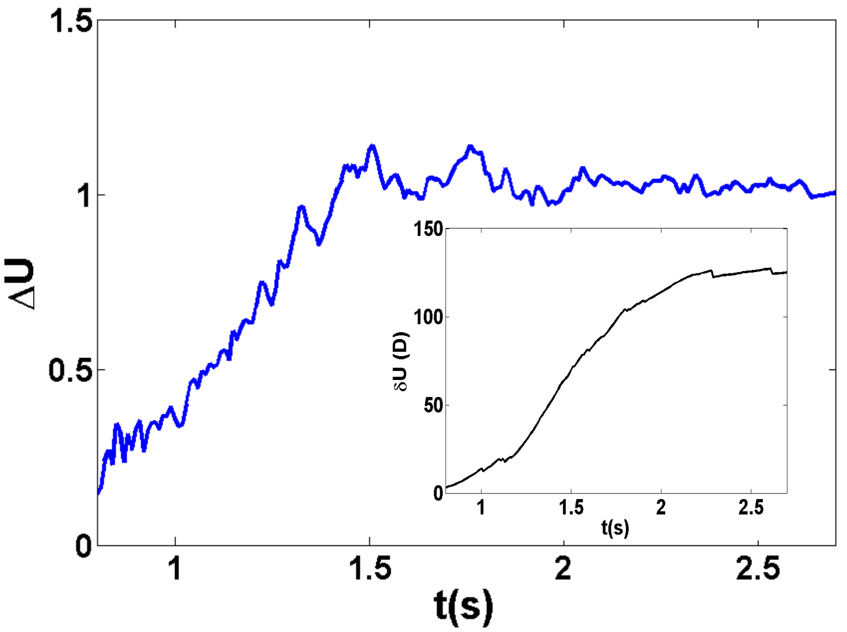

FIG. 1: The modulus of the first global Lyapunov vector $\Delta U(t)$ in the phase space that includes the contribution of the velocity as a function of time t near unjamming. We normalize $\Delta U(t)$ by $\Delta U(t) = \sqrt{\frac{\sum_i \delta u_i(t)^2}{\delta U(t)^2_{max}} + \frac{\sum_i v_i(t)^2}{V(t)^2_{max}}}$. In comparison, the inset shows $\delta U(t) = \sqrt{\sum_i \delta u_i(t)^2}$ with no inclusion of the velocity. The units, D, of δU is the average diameter of the disk.

---

[0] *Correspondence to [jiezhang2012@sjtu.edu.cn].

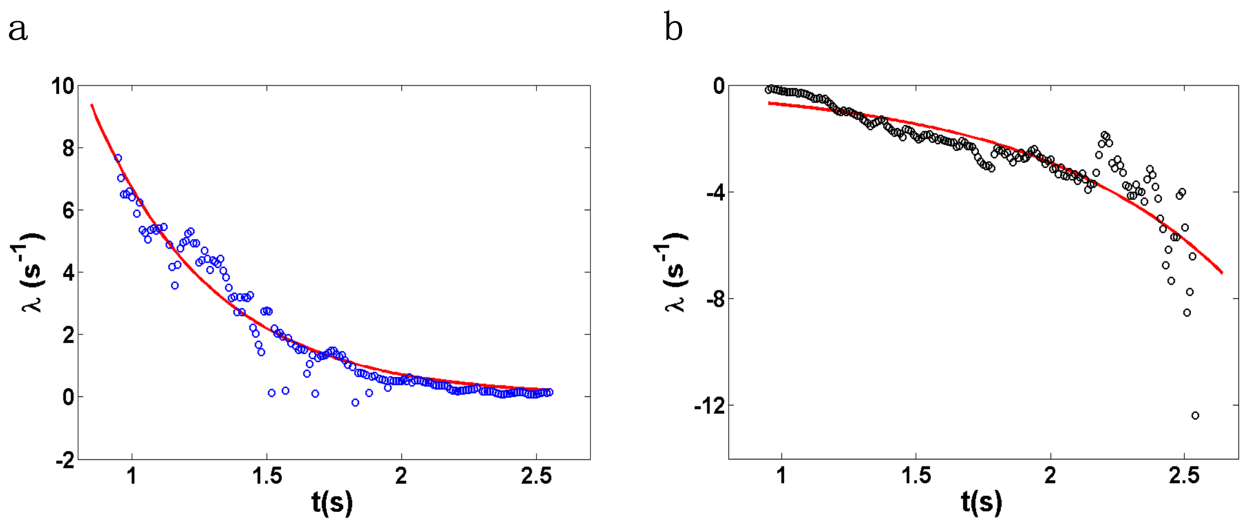

FIG. 2: The global Lyapunov exponent λ in (a) the unjamming regime and in (b) the jamming regime. In each panel, the dashed line extends the calculation of λ into the whole range of the avalanche process. Red solid lines are fit using the function $\lambda(t) = \alpha e^{\beta t}$.

TABLE I: Various fitting coefficients related to the Lyapunov exponent λ.

avalanche number	λ_{unj}	λ_{jam}	(α, β)	(α, β)
$n_0 = 1$	12.1	-21.0	16.2, -1.9	-0.0046, 3.4
$n_0 = 2$	9.3	-7.0	62.6, -2.2	-0.18, 1.3
$n_0 = 3$	11.1	-8.0	66.9, -2.3	-0.29, 1.3
$n_0 = 4$	6.7	-6.5	13.7, -1.5	-0.30, 1.3
$n_0 = 5$	6.2	-8.3	16.3, -1.2	-0.025, 2.3
$n_0 = 6$	5.5	-10.2	400.0, -1.6	-0.00074, 2.1
$n_0 = 7$	11.2	-11.5	150.4, -4.7	-0.10, 2.8
$n_0 = 8$	15.4	-6.3	113.3, -4.4	-0.30, 1.3
$n_0 = 9$	9.3	-4.7	43.0, -2.3	-0.38, 1.1
$n_0 = 10$	8.0	-3.84	871.4, -2.6	-0.0013, 2.4
mean	9.5	-8.8	175.4, -2.5	-0.16, 2.0

II. THE GLOBAL LYAPUNOV EXPONENTS

To characterize the rate of divergence (respectively, convergence) of the system from a fixed point near the unjamming (respectively, jamming) transition, we define the global Lyapunov exponent as $\lambda = \frac{1}{\Delta t} ln(\frac{\delta U(t)}{\delta U(t-\Delta t)})$, with $\Delta t = 0.01s$ as shown in Fig. 2. Here λ measures the average exponential growth rate of the global Lyapunov vector δU (defined in the main text) over a short time $\Delta t = 0.01s$ which is chosen to be the inverse of the frame rate of the imaging. As shown in the figure, except for a few sharp spikes, most likely due to noises, λ is positive in the unjamming regime and becomes negative in the jamming regime. To make a comparison among all runs, we fit the curves in Fig. 2(a-b) with the function of $\lambda(t) = \alpha e^{\beta t}$.Table I summarizes the fitting coefficients α, β of different runs of the unjamming and jamming regimes. Note that in each run, the starting point of an avalanche is not zero because experimentally in order to record the whole sequence of the avalanche process, we often start with an arbitrary time shift before the actual starting of the avalanche. This is reflected in the wide range of fluctuation of the fitting parameter α. We may shift the time t to t' such that the avalanche starts at $t' = 0$ and the fitting function becomes $\lambda(t') = \lambda_{unj} e^{\beta t'}$ for panel (a); we may also shift the time t to t'' such that the avalanche ends at $t'' = 0$ and $\lambda(t'') = \lambda_{jam} e^{\beta t''}$ for panel (b). From Table I, for all runs the values of λ_{unj} are positive and λ_{jam} are negative, which is robust. For different runs, λ_{unj} or λ_{jam} are within the same order of magnitude despite the fluctuations. Compared to the mean values, individual values could reach twice larger or smaller.

III. THE MEAN-FIELD MODEL

In order to explain the spatial correlations between δu and v and the temporal correlations between δU, η and V in the unjamming regime, we propose a mean-field model based on the dynamics of $v(t)$ and $N(t)$.

(a) The spatial correlation between $\delta u_i(t)$ and $v_i(t)$

As discussed in detail in the main text, the velocity $v_i(t)$ of a given particle i in the avalanche can be divided into three regimes – the exponential regime, the inertial regime, and the frictional regime. If the particle is in the early time range of the unjamming regime, its velocity $v_i(t)$ can be simply modeled as two parts – an exponential part followed by an inertial part so that

$$v_i(t) = \begin{cases} v_0 e^{at} & 0 < t < t_0 \\ bt & t_0 < t < t_1 \end{cases}$$

where t_0 (respectively, t_1) is the starting point of the inertial (respectively, the frictional) regime and $v_0 = 2.5D/s$, with D the average diameter of the disk, is the minimum resolvable velocity in the experiment. Note that in the figure 4(c) of the main text, for time $t < 1.4s$ the fraction of particles in the frictional regime is less than ten percent and thus the exclusion of the frictional regime in the above model is statistically valid. Since the ideal trajectory contributes negligibly compared with the real one to the first Lyapunov vector δu_i, we can simply approximate δu_i using its actual displacement in the real space, which allows us to obtain δu_i by integration:

$$\delta u_i(T) = \int_0^T v_i(t)dt$$

$$= \begin{cases} v_0(e^{aT}-1)\frac{1}{a} \approx v_0 e^{aT}\frac{1}{a} = \frac{1}{a}v_i(T). & 0 < T < t_0 \\ v_0(e^{at_0}-1)\frac{1}{a} + \frac{1}{2}b(T^2-t_0^2) \approx \frac{1}{2}bT^2 = \frac{1}{2}\frac{v_i^2(T)}{b}. & t_0 < T < t_1 \end{cases}$$

So $\delta u_i(T)$ and $v_i(T)$ are spatially correlated in the leading order whether $v_i(t)$ is in the exponential regime or in the inertial regime.

(b) The temporal correlations between δU, η, and V

In the unjamming regime, individual moving particle can trigger particles of the neighbouring regions along its pathway – mainly at the upstream of the inclination – to lose stability, causing a cascade of local unjamming transitions as the whole system is rapidly diverging from the unstable fixed point. As a result, $N(t)$ increases substantially, and assuming a constant growth rate ξ we have

$$N(t) = e^{\xi t}$$

which is consistent with the inset of the figure 2(b) of the main text, with $\xi = 7s^{-1}$.

To find $\delta U(t)$, we compute the convolution of $N(t)'$ and $\delta u_i(t)$ by assuming each mobile particle behaves similarly, so that

$$\delta U(T) = \int_0^T N(t)'\delta u_i(T-t)dt = \int_0^T \xi e^{\xi t}\delta u_i(T-t)dt$$

$$= \begin{cases} \frac{\xi v_0}{a(\xi-a)}(e^{\xi T}-e^{aT}), & 0 < T < t_0 \\ C_1 e^{\xi T} + C_2 T^2 + C_3 T + C_4, & t_0 < T < t_1 \end{cases}$$

with

$$C_1 = \left(\frac{b}{2}t_0^2 + \frac{b}{\xi}t_0 + \frac{b}{\xi^2}\right)e^{-\xi t_0} + \frac{\xi v_0}{a(\xi-a)}[1-e^{(a-\xi)t_0}]$$

$$C_2 = -\frac{b}{2}, C_3 = -\frac{b}{\xi}, C_4 = -\frac{b}{\xi^2}$$

Taking derivative of $\delta U(T)$, we have $\eta(T)$ as follows

$$\eta(T) = \delta U(T)' = \begin{cases} \frac{\xi v_0}{a(\xi-a)}(\xi e^{\xi T} - a e^{aT}), & 0 < T < t_0 \\ D_1 e^{\xi T} + D_2 T + D_3, & t_0 < T < t_1 \end{cases}$$

TABLE II: Various parameters used in the mean field model.

avalanche number	t_0	t_1	a	b	ξ
$n_0 = 1$	0.059	0.53	23.4	20.4	4.0
$n_0 = 2$	0.17	0.54	10.6	73.5	7.0
$n_0 = 3$	0.079	0.21	16.7	70.7	7.7
$n_0 = 4$	0.053	0.28	15.0	78.9	5.9
$n_0 = 5$	0.036	0.12	56.2	193.7	4.1
$n_0 = 6$	0.058	0.22	15.3	68.7	4.3
$n_0 = 7$	0.056	0.20	16.1	84.5	12.6
$n_0 = 8$	0.043	0.16	24.1	107.8	5.2
$n_0 = 9$	0.062	0.29	15.6	86.3	11.1
$n_0 = 10$	0.030	0.11	49.9	246.7	2.9
mean	0.065	0.22	20.2	85.9	5.4

with

$$D_1 = (\frac{b}{2}\xi t_0^2 + bt_0 + \frac{b}{\xi})e^{-\xi t_0} + \frac{\xi^2 v_0}{a(\xi - a)}[1 - e^{(a-\xi)t_0}]$$

$$D_2 = -b, D_3 = -\frac{b}{\xi}$$

Similarly, $V(T)$ of the system is the convolution of $N(t)'$ and $v_i(t)$:

$$V(T) = \int_0^T N_i(t)' v_i(T-t)dt = \int_0^T \xi e^{\xi t} v_i(T-t)dt$$

$$= \begin{cases} \frac{\xi v_0}{\xi - a}(e^{\xi T} - e^{aT}), & 0 < T < t_0 \\ E_1 e^{\xi T} + E_2 T + E_3, & t_0 < T < t_1 \end{cases}$$

with

$$E_1 = (bt_0 + \frac{b}{\xi})e^{-\xi t_0} + \frac{\xi v_0}{\xi - a}(1 - e^{-\xi t_0}), E_2 = -b, E_3 = -\frac{b}{\xi}.$$

In order to test the validity of our theoretical model, we calculate δU, V and η using the expressions introduced in the main text with the typical parameters determined from the experiment: $t_0 = 0.18s, t_1 = 0.55s, a = 10.6s^{-1}, b = 73.5D/s^2, \xi = 7s^{-1}$. Except ξ which is estimated from the figure 2(b) of the main text, the values of the other parameter are obtained from the statistical averages according to their probability distribution functions shown in Fig. 3. The results are plotted in Fig. 4, showing strong correlations between δU, V, and η, which is less obvious from the direct examination of their expressions. So the correlation between these three macroscopic variables can be well captured by our model. There is a small discontinuity near the beginning of the curve in Fig. 4 due to the simplification in our model where an effective coefficient b is applied to approximate the linear relation of $v_i(t)$ after t_0, causing a discontinuity in v_i and subsequently all the derived macroscopic variables at t_0. In table II, we have listed all the five parameters used in the above mean field model for different runs. The values of each set of parameters vary from run to run with fluctuations up to a few times larger or smaller compared to the mean value. Note that the formulation of this model is based on two important pieces of information from experimental measurements: (1) the velocity of individual particles can be simply divided into three regimes – the exponential, inertial and frictional regimes, which allows us to distinguish the jamming and unjamming regimes; (2) the number of the moving particles $N(t)$ increases (respectively, decays) exponentially in the unjamming (respectively, jamming) regime. Using the typical parameter values, in the range of $t_0 < T < t_1$, the exponential terms $C_1 e^{\xi T}$, $D_1 e^{\xi T}$, and $E_1 e^{\xi T}$ make the dominant contributions in the expressions of δU, η, and V, as plotted in Fig. 5. Hence it is the exponential form of $N(t)$ that contributes the most to the linear correlations seen above.

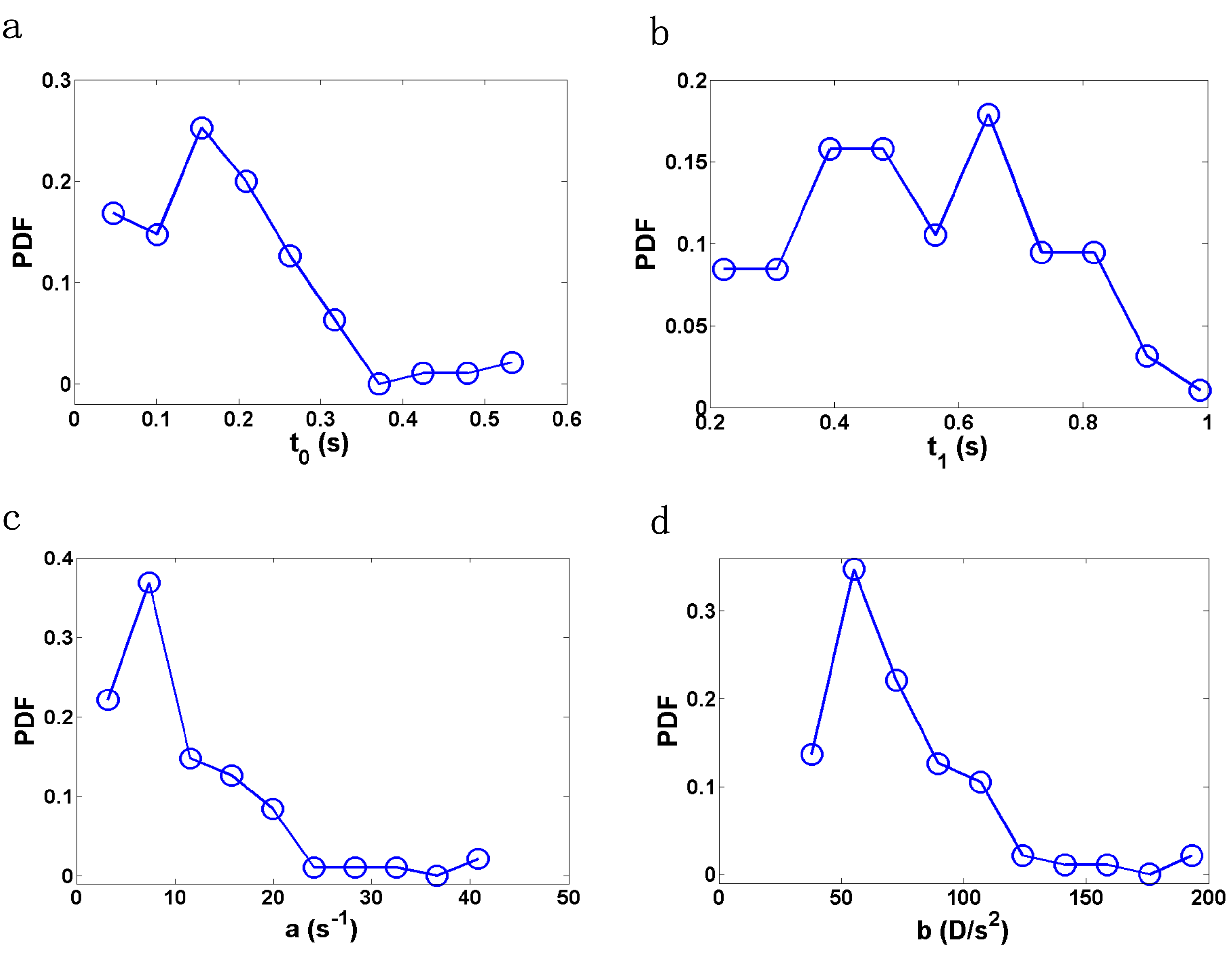

FIG. 3: The probability distribution functions of various parameters defined in the theoretical model, including (a) t_0, (b) t_1, (c) a, and (d) b, respectively. In panel (d), in the units of b, D is the average diameter of the disk.

---

[1] Pine, D.J., Gollub, J.P., Brady, J.F. and Leshansky, A.M. Chaos and threshold for irreversibility in sheared suspensions, *Nature* **438**, 7070, 997–1000 (2005).

[2] Corte, L., Chaikin, P.M., Gollub, J.P. and Pine, D.J. Random organization in periodically driven systems, *Nature Phys.* **4**, 5, 420–424 (2008).

[3] Keim, N.C. and Arratia, P.E. Mechanical and microscopic properties of the reversible plastic regime in a 2D jammed material, *Phys. Rev. Lett.* **112**, 2, 028302 (2014).

[4] Regev, I., Lookman, T. and Reichhardt, C. Onset of irreversibility and chaos in amorphous solids under periodic shear, *Phys. Rev. E* **88**, 6, 062401 (2013).

[5] Slotterback, S. et al. Onset of irreversibility in cyclic shear of granular packings, *Phys. Rev. E* **85**, 2, 021309 (2012).

[6] Amon, D.L., Niculescu, T. and Utter, B.C. Granular avalanches in a two-dimensional rotating drum with imposed vertical vibration, *Phys. Rev. E* **88**, 1, 012203 (2013).

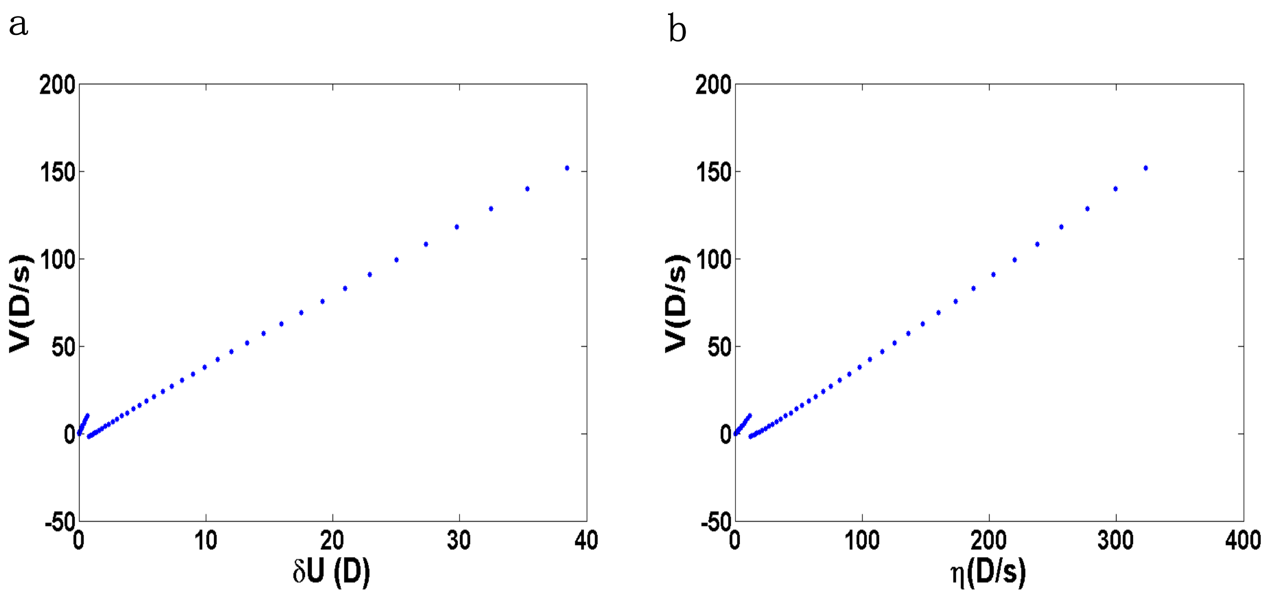

FIG. 4: Results calculated using our theoretical model: (a) δU versus V inside the unjamming regime (b) η versus V inside the unjamming regime. Here in the units of δU, η, and V, D is the average diameter of the disk.

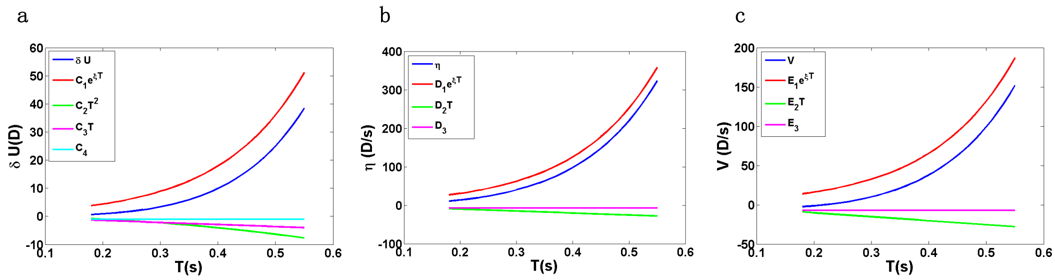

FIG. 5: (a) δU versus time t (blue curve) along with the time curves of the four separate terms in the expression of δU. (b) η versus time t (blue curve) along with the time curves of the three separate terms in the expression of η. (c) V versus time t (blue curve) along with the time curves of the three separate terms in the expression of V. All results here are calculated using the theoretical model. Here in the units of δU, η, and V, D is the average diameter of the disk.
